# Bone mineral density measurements in vertebral specimens and phantoms using dual-layer spectral computed tomography

**DOI:** 10.1038/s41598-017-17855-4

**Published:** 2017-12-13

**Authors:** Kai Mei, Benedikt J. Schwaiger, Felix K. Kopp, Sebastian Ehn, Alexandra S. Gersing, Jan S. Kirschke, Daniela Muenzel, Alexander A. Fingerle, Ernst J. Rummeny, Franz Pfeiffer, Thomas Baum, Peter B. Noël

**Affiliations:** 1 Department of Radiology, Klinikum rechts der Isar, Technical University of Munich, Munich, Germany; 2 Department of Neuroradiology, Klinikum rechts der Isar, Technical University of Munich, Munich, Germany; 3 Physics Department & Munich School of BioEngineering, Technical University of Munich, Munich, Germany

## Abstract

To assess whether phantomless calcium-hydroxyapatite (HA) specific bone mineral density (BMD) measurements with dual-layer spectral computed tomography are accurate in phantoms and vertebral specimens. *Ex-vivo* human vertebrae (n = 13) and a phantom containing different known HA concentrations were placed in a semi-anthropomorphic abdomen phantom with different extension rings simulating different degrees of obesity. Phantomless dual-layer spectral CT was performed at different tube current settings (500, 250, 125 and 50 mAs). HA-specific BMD was derived from spectral-based virtual monoenergetic images at 50 keV and 200 keV. Values were compared to the HA concentrations of the phantoms and conventional qCT measurements using a reference phantom, respectively. Above 125 mAs, errors for phantom measurements ranged between −1.3% to 4.8%, based on spectral information. In vertebral specimens, high correlations were found between BMD values assessed with spectral CT and conventional qCT (r ranging between 0.96 and 0.99; p < 0.001 for all) with different extension rings, and a high agreement was found in Bland Altman plots. Different degrees of obesity did not have a significant influence on measurements (P > 0.05 for all). These results suggest a high validity of HA-specific BMD measurements based on dual-layer spectral CT examinations in setups simulating different degrees of obesity without the need for a reference phantom, thus demonstrating their feasibility in clinical routine.

## Introduction

Osteoporosis is estimated to affect 28 million patients in the European Union with increasing prevalence, due to the aging population^1,2^. Reducing bone stability and thus increasing the risk of fractures, osteoporosis substantially adds to morbidity and mortality not only of the elderly, but also of younger subjects with impaired bone metabolism as present in patients with cancer or eating disorders^3,4^. However, up to 70% of eligible women and far more men do not undergo bone mineral density (BMD) screening with dual-energy x-ray absorptiometry (DXA), the clinical standard^5,6^, although BMD is a predictor for future fracture risk and all-cause mortaliy^7,8^. Subsequently, only 10–22% of patients receive adequate therapy for osteoporosis^1,2,9^. In addition to this, DXA values have shown to be affected by several confounding factors, e.g. spondylosis, patient size, and vascular calcifications, likely causing DXA to correctly diagnose osteoporosis in only about 44% of women and 21% of men with prevalent osteoporotic fractures^10^.

qCT allows for volumetric BMD measurements, commonly applied to the spine, and calibrated to a reference phantom with known hydroxyapatite concentrations, may outperform DXA-based BMD measurements^11^, and also allows more advanced models for fracture risk assessment such as finite element analysis (FEA)^12–14^. In order to reduce radiation doses, ongoing research also focuses on how to opportunistically use multidetector computed tomography (MDCT) data obtained for various reasons, e.g. for morphologic imaging, disease staging for osteoporosis screening or therapy monitoring^15^. For example, BMD-equivalent values derived from MDCT have previously been used to predict screw loosening in subjects undergoing spondylodesis^16^ and incidental osteoporotic vertebral fractures^17^, and to assess the fracture risk in subjects with inflammatory bowel disease and prostate cancer^18,19^. However, there are limitations to all CT-based BMD measurements, since they can be affected by scan parameters such as different tube voltages, the use of intravenous contrast medium, the fat fraction within vertebral bone marrow, and beam hardening or scatter artefacts^20–24^.

One CT technique gaining more and more attention is acquiring spectral information using dual-energy CT (DECT). There are different approaches to exploit the spectral information such as fast kV-switching or using two x-ray sources with different characteristics, known as dual-source CT^25–27^. Recently a new technique with one x-ray tube and two different detector layers mounted upon each other and absorbing different energy spectra of the polychromatic X-ray spectrum, known as dual-layer spectral CT, was introduced^27,28^. While DECT requires the pre-selection of specific dual-energy CT protocols, spectral CT allows for routinely reconstructing spectral information without the use of a specific protocol. Those methods enable the estimation of object composition by exploiting material- and energy-dependent x-ray absorption of various materials. For example, the quantification of iodine is already commercially available and can be applied to clinical routine. DECT has shown to be feasible for volumetric BMD assessment of the lumbar spine using DXA as a standard of reference^29^. A recent study reported a high accuracy of density measurements in phantoms with known hydroxyapatite (HA) concentrations^30^, however, to our best knowledge, the validity of HA-specific BMD measurements has not been assessed previously in vertebral specimens.

Aims of this study therefore were to (i) validate spectral CT based, HA-specific mineral density quantification with a phantom with known HA densities as reference standard, and (ii) to validate spectral CT based BMD quantification in vertebral specimens with qCT as standard reference; both in a setup with an anthropomorphic phantom simulating different degrees of obesity.

## Results

### Radiation doses, SNR and CNR

CT Dose Index (CTDI_vol_) ranged between 1.2 mGy at 10 mAs and 63.1 at 500 mAs. At 500 mAs, the signal-to-noise was 29.0 and the contrast-to-noise was 32.6 (Table 1).

**Table 1:** CT dose indices (CTDI_vol_), estimated effective doses (ED), signal-to-noise (SNR), and contrast-to-noise (CNR) for phantom measurements at different exposure levels.

Exposure	CTDI_vol_ (mGy)	ED (mSv)	SNR	CNR
500 mAs	63.1	4.2	29.0	32.6
250 mAs	30.7	2.1	21.2	23.9
125 mAs	15.3	1.1	15.8	17.8
50 mAs	6.1	0.4	11.5	12.8
10 mAs	1.2	0.1	8.8	9.6

### Spectral-based BMD quantification in phantoms with known HA densities

Measurements of the four known HA densities of a dedicated phantom produced accurate values, with measurement errors between 0.5% and 4.8% of the specified densities for scans acquired with 125 mAs, which are comparable to the radiation exposures used in most clinical examination protocols, or more (Table 2). At our lowest exposure level, measurement errors increased up to 14.8% (lowest HA concentration assessed with 38 mAs).

**Table 2:** BMD measurements based on spectral CT compared to a phantom containing four known concentrations of calcium hydroxyapatite (HA) (100, 200, 400, and 800 mg/cm^3^).

Measurement differences:	Phantom HA concentrations:							
	800 mg/cm^3^		400 mg/cm^3^		200 mg/cm^3^		100 mg/cm^3^	
	mg/cm^3^	%	mg/cm^3^	%	mg/cm^3^	%	mg/cm^3^	%
Exposure:								
1000 mAs	+1.2	+0.15%	−2.3	−0.56%	−1.5	−0.71%	+2.6	+2.58%
500 mAs	−0.3	−0.03%	−3.5	−0.87%	−1.3	−0.61%	+3.5	+3.49%
250 mAs	+1.5	+0.19%	−3.2	−0.79%	−2.6	−1.26%	+3.0	+2.99%
125 mAs	+13.2	+1.63%	+2.0	+0.51%	+3.1	+1.49%	+4.8	+4.79%
50 mAs	+43.0	+5.32%	+24.2	+5.98%	+13.8	+6.62%	+11.2	+11.22%
38 mAs	+53.1	+6.56%	+26.7	+6.58%	+18.4	+8.83%	+14.8	+14.84%

Positive differences indicate higher BMD measurement values compared to known HA densities as specified by the phantom manufacturer.

### Spectral-based BMD quantification in vertebral specimens

**Figure 1:**
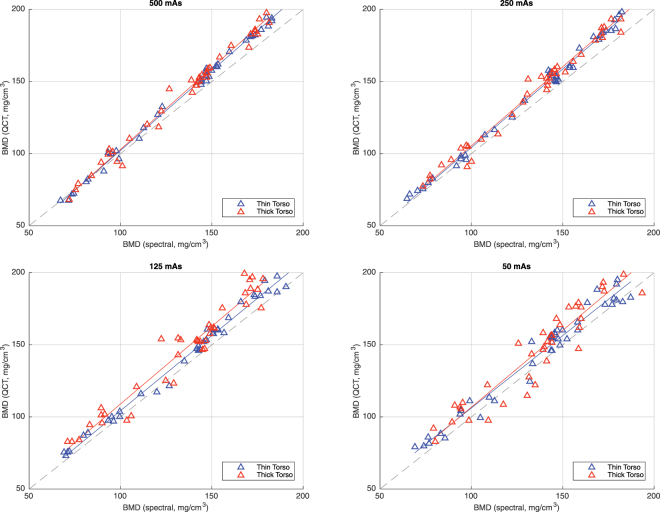
BMD values based on spectral information and qCT, respectively, shown separately for 500, 250, 125 and 50 mAs. Plots indicate high correlations (r > 0.96 for all) Triangles indicate measurement pairs for a higher (red) and lower (blue) simulated degree of obesity.

While BMD measurements in mid-vertebral specimens derived from conventional qCT ranged between 141 and 144 mg/cm^3^, estimation of HA density based on spectral data ranged between 134 and 136 mg/cm^3^, scanned at 50 mAs or more (Table 3). All spectral estimations of HA density acquired with an exposure of 50 mAs or more were highly correlated with those estimated using qCT (r > 0.96 for all; Fig. 1). No statistical significant differences were found between the spectral and qCT measurements from 50 mAs to 500 mAs (p > 0.02), however, at 10 mAs significant differences were found between qCT and spectral CT values.

**Table 3:** BMD measurements in vertebral specimen based on spectral CT and conventional qCT, as well as mean differences between measurements and correlation coefficients, listed separately for different exposure levels.

Exposure:	qCT	Spectral CT	Mean Difference	Correlation (Pearson’s r)	t-test (p-value)
500 mAs	141.2 ± 40.3	134.5 ± 29.1	−6.7 ± 5.7	1.00	0.27
250 mAs	141.8 ± 46.9	133.9 ± 31.1	−7.9 ± 5.5	0.99	0.18
125 mAs	143.6 ± 55.5	135.0 ± 33.8	−8.6 ± 7.6	0.98	0.14
50 mAs	144.1 ± 66.1	136.4 ± 36.6	−7.7 ± 9.6	0.96	0.15
10 mAs	122.6 ± 71.3	168.9 ± 59.3	+46.3 ± 75.2	0.26^a^	0.00^b^

Mean values ± standard deviation (SD) and mean differences ± SD are given in mg/cm^3^.

^a^Indicates a statistically not significant correlation (p > 0.05). ^b^Indicates a significant difference in the paired-samples t-test (p < 0.02).

**Figure 2:**
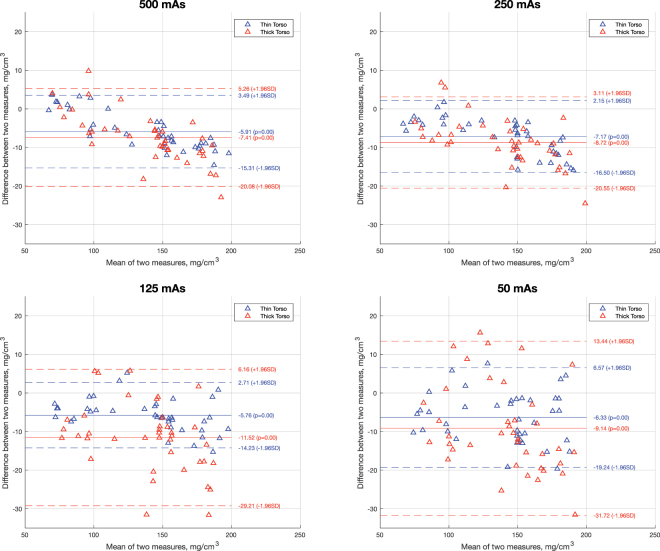
Bland-Altman plots showing the means versus the difference of BMD measurements derived from spectral CT and qCT separately for different tube exposures (500, 250, 125 and 50 mAs) as well as for a higher (red) and lower (blue) simulated degree of obesity, respectively. Solid lines indicate mean BMD differences; the dotted lines indicate mean difference ±1.96 SD. With most values within the range of the mean difference ±1.96 SD, Bland-Altman plots indicate a high agreement of measurements^42^.

In addition, agreement between BMD results based on spectral CT and qCT were assessed with a Bland-Altman plot (Fig. 2 **)**. At 50 mAs and above, mean differences were between ±1.96 SD in most cases, indicating a good agreement between both measurements. At 50 mAs and more, measurements from spectral CT were consistently lower than qCT measurements by a small margin (mean differences, 6.7–8.6 mg/cm^3^ for 50 mAs and above; Table 3).

When comparing BMD results from spectral CT acquired with two different setups simulating two different degrees of obesity, no significant differences were found (Table 4, p > 0.02).

**Table 4:** Mean differences of BMD measurements based on spectral CT in the two different setups simulating different degrees of obesity.

Exposure:	Mean differences between degrees of obesity	t test (p-value)
500 mAs	−0.4 ± 7.3	0.96
250 mAs	±1.1 ± 8.4	0.89
125 mAs	−2.4 ± 10.9	0.76
50 mAs	−0.1 ± 9.5	0.99
10 mAs	−15.2 ± 83.7	0.39

Mean differences ± standard deviation (SD) are given in mg/cm^3^. P-values calculated using the paired-samples t-test.

In linear regression models with the simulated degree of obesity as an additional independent variable, significant correlations were found between qCT- and spectral CT-based BMD measurements for all exposure levels, with high correlation coefficients for 50–500 mAs (Betas; 500 mAs: 0.99; 250 mAs: 0.99; 125 mAs: 0.97; 50 mAs: 0.98) and only a moderate correlation for 10 mAs (Beta, 0.27). For 50 mAs, a small but significant association between the degree of obesity and the BMD measurements based on spectral CT were found (Beta, 0.08; p < 0.001). For all other exposures, the degree of obesity was not significantly associated with the measurements (p > 0.001).

## Discussion

In our study, HA-specific density measurements based on spectral data obtained with a dual-layer spectral CT showed high accuracy when performed on a phantom with different known HA concentrations in scans with radiation exposures comparable to clinical protocols. Moreover, phantomless HA-specific spectral BMD measurements showed high correlations and good agreement with conventional qCT-based measurements, which are the current standard of reference for assessing volumetric BMD. To our best knowledge, this is the first time the potential value of phantomless BMD measurements based on dual-layer spectral CT scans has been assessed.

While osteoporosis is associated with a substantial morbidity and mortality, the condition is underdiagnosed and thus undertreated^1,2,5,6,31,32^. Therefore, patients would highly benefit from a reliable opportunistic BMD assessment and thus fracture risk assessment with non-dedicated imaging data.

Several approaches for opportunistic measurements based on CT data have been investigated recently, such as obtaining BMD information from contrast-enhanced routine abdominal examinations^17,33^ and CT colonography^18^ as well as non-contrast enhanced or post-myelography CT examinations^16^, and BMD-equivalent values derived from those examinations have shown to highly correlate with values obtained from phantom-calibrated qCT examinations. However, there are limitations to these opportunistic measurements: First, patient size potentially affects BMD values^34^. A larger abdomen volume is able to alter the x-ray spectrum received at the detector, increasing the possibility of beam hardening. The absorption of bone mineral is energy-dependent and thus when estimating BMD from the full spectrum, a HA calibration phantom should be used which is often not placed in the scanner couch during routine scans. Second, it has been shown that the application of intravenous contrast agents substantially affects BMD measurements, presumably due to iodine altering attenuation in measurement ROIs within the vertebrae^23,24^. It can be assumed, though, that a spectral-based HA-specific measurement is more robust against a possible bias by other materials affecting attenuation. Material decomposition based on spectral information is already commercially available, e.g. iodine concentration can be quantified and used for the imaging of myocardial perfusion or pulmonary embolism^35^. However, while previous studies reported BMD quantification in cadaveric specimen and patients based on dual-source spectral CT examinations to be feasible^29,36^, material decomposition and thus HA-specific quantification were not considered in these previous studies. This is noteworthy since it has been shown that marrow adipose tissue may add a substantial bias to CT-based trabecular BMD measurements^37^.

Monoenergetic images can be generated not only based on dual-layer spectral CT, but also based on other DECT approaches such as set-ups generating two different x-ray spectra^38^. However, such systems require pre-specified dual-energy scan protocols using both x-ray spectra. In addition, in case of dual-source CT, two x-ray sources generate scatter radiation on each other, which needs to be corrected for, as shown previously^39^. Moreover, due to the compact design and the perpendicular set-up of systems with dual x-ray sources, one of the energies has a smaller field of view, which limits spectral analysis and image quality for larger patients. Finally, spectral information and thus material decomposition can only be post-computed in image space — in contrast to dual-layer spectral CT, which allows for direct material decomposition in projection space — which reduces the accuracy of the material decomposition and the effectiveness of beam-hardening corrections.

Just recently van Hamersvelt *et al*. investigated BMD quantification in phantoms with known HA-concentrations based on examinations with a dual-layer spectral CT system identical to the one used in this study, describing measurements to be highly accurate^30^. In their study, the authors postulated exact concentrations of water and HA within resin phantoms and applied attenuation profiles from the NIST database and estimated BMD values. However, fitting tabulated database values may be inaccurate for several reasons: Firstly, in a setup with a clinical scanner, virtual monoenergetic are not equivalent and only approximations to actual monoenergetic measurements. Secondly, the specific detector response and the spectral decomposition algorithm need to be taken into account, as they may differ from system to system and particularly if reference values were transferred from a different DECT approach. In contrast to the mentioned study using NIST database attenuation profiles, we modelled the full imaging chain for our specific setup by acquiring calibration measurements using a phantom with known HA concentrations. Using this approach, we were able to create scanner-specific attenuation profiles, which enabled us to overcome software and hardware inconsistencies, assuming there is proprietary information about the system used in this study that is unknown. In the study of van Hamersvelt *et al*. measurement errors ranged between 0.5 and 5.2% for 100–200 mAs, which is comparable to the errors of 0.0 to 4.8% we observed in our analyses for exposures of 125 mAs (a tube exposure typically used in clinical protocols) and above. Moreover, this is in accordance with the study by Hofmann *et al*. presenting a three-material decomposition method for BMD quantification on DECT, finding a mean measurement error of about 3.5% over all HA concentrations^38^.

Interestingly, Hofmann *et al*. found that measurements of known HA densities obtained from qCT overestimated actual known densities, which also is in accordance with our findings that qCT values were slightly higher than values from spectral CT. As discussed before, this may indicate that HA-specific measurements based on spectral analyses may be more accurate than qCT measurements^30,38^.

Measurement errors at 50 and 38 mAs were more substantial in lower HA concentrations (14.8% for 100 mg/cm^3^ as assessed with 38 mAs). This may be caused by the fact that a lowered tube current decreases the number of photons emitted, thus influencing the final number of photons reaching the layers of the detector and ultimately the accuracy of spectral signal and BMD measurements. This is a known limitation of all dual-layer detectors and should be taken into account when assessing the clinical value of dedicated ultra-low-dose CT protocols. Other means of dose reduction such as sparse sampling should be considered^40^.

BMD measurements in cadaveric vertebral specimens specific for HA based on spectral CT imaging showed high correlations and agreement with measurements obtained using phantom-calibrated qCT as standard of reference. Correlations were significant based on scans with 50–500 mAs, with correlation coefficients increasing with increasing mAs. This suggests that HA-specific BMD quantification is valid in vertebral specimens, and should also be feasible *in-vivo*.

Note, when comparing BMD measurements in vertebral specimens based on spectral CT in setups simulating two different degrees of obesity, we did not find any significant differences. Moreover, in linear regressions, no significant associations were found between degrees of obesity and BMD based on spectral CT, which may be particularly beneficial to obese patients, in which therapeutic decisions should be based on accurate measurements as well. It is noteworthy since DXA as the current clinical standard and qCT have been reported to be significantly affected by body composition and mAss^8,11,14,15^, and it suggests that spectral CT may be more robust to such external confounders.

Our study has limitations. First, we used monoenergetic images at 50 and 200 keV to assess HA characteristics and concentrations. However, in a dual-layer setup, we can assume that two monoenergetic levels can represent the complete spectrum information obtained in the scanner. Second, we assessed both known HA concentrations in phantoms and BMD in vertebral specimens in standardized setups, including the two different degrees of obesity simulated by an anthropomorphic phantom with two different extension rings; however, other factors that have been described to affect the BMD quantification such as different tube voltages, the use of intravenous contrast medium, the fat fraction within vertebral bone marrow were not taken into account in this setup^20–24^. Therefore, further studies are necessary assessing clinical patient scans with different scan protocols. Finally, plain thresholding was applied to define HA-containing pixels, with a 20 HU threshold. Since in this study in both, the setup with phantoms with known HA concentrations as well as the setup with vertebral specimens, only HA and water were present in the ROIs, therefore different threshold values (assessed for 20 to 100 HU) did not measurably affect final BMD values. However, in future *in-vivo* studies additional materials such as iodine may be present and need to be taken into account and different thresholding techniques may be needed in order to reliably identify HA.

In summary, we have shown that known HA concentrations can be quantified accurately by using spectral CT imaging information obtained with a dual-layer spectral CT scanner. In addition, high correlations and agreement between HA-specific BMD measurements in vertebral specimens and phantom-calibrated qCT measurements in the same specimens in setups simulating different degrees of obesity were found. This finding suggests that phantomless BMD quantification based on dual-layer spectral CT imaging is feasible and may be applied in clinical studies.

## Methods

### Specimens and phantoms

Institutional review board approval was obtained prior to this study (Ethikkommission der medizinischen Fakultät, Technical University of Munich, Germany).

**Figure 3:**
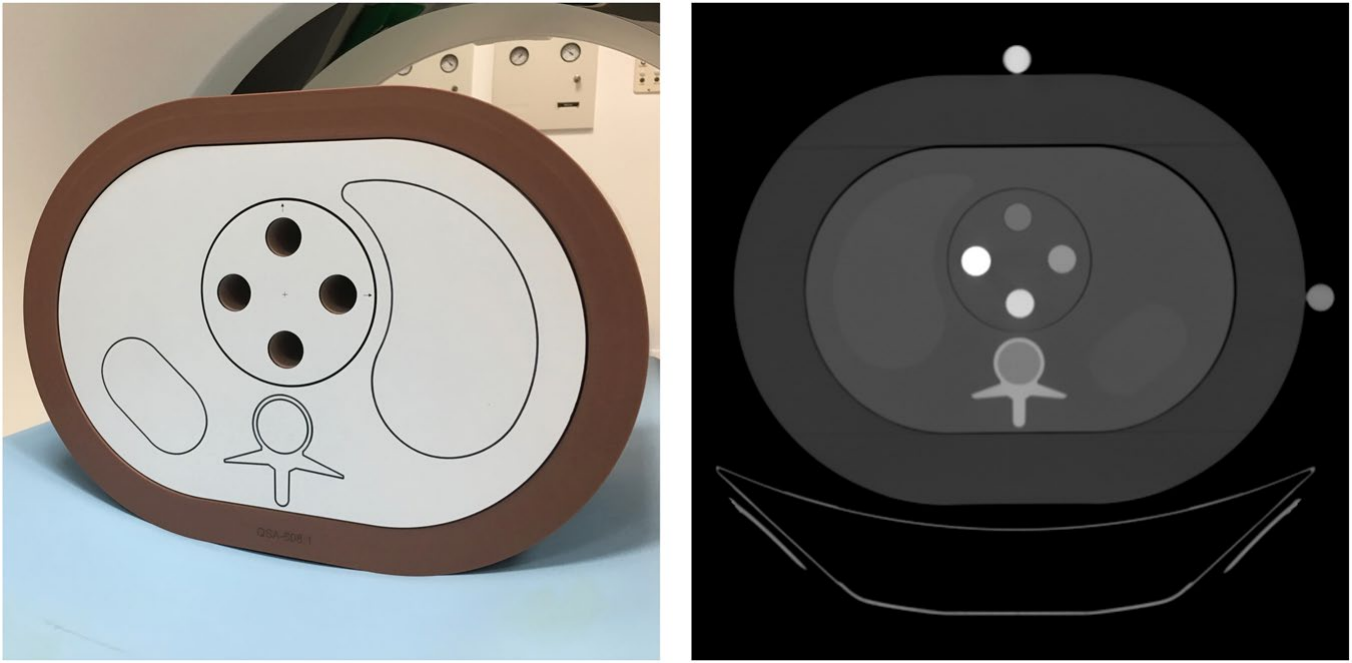
Anthropomorphic phantom with the smaller (photograph; left) and larger (CT scan; right) extension ring simulating different degrees of obesity containing a four-bore CT water insert, in which four cylinders with known HA densities were placed (as shown in the CT scan; right). For the vertebral specimen scans, the insert was replaced with a water insert containing the specimens (Fig. 4).

Four cylindrical phantoms (QRM) with known calcium hydroxyapatite (HA) concentrations were examined in an anthropomorphic phantom (Anthropomorphic Abdomen Phantom, QRM, Möhrendorf, Germany; Fig. 3). The exact HA concentrations were 809, 405, 209 and 100 mg/ml. The composition of the phantoms was CT water (0 HU, 80–120 kV) and HA. The base materials of the abdomen phantom were tissue equivalent resin (about 35 to 50 HU at 120 keV) imitating muscle and liver, etc. The axial diameter was 300 × 200 mm.

In a second setup, 13 mid-vertebral specimens harvested from human donors without history of pathological bone changes other than osteoporosis were examined in this study. The donors had dedicated their bodies for educational and research purposes to our institution and gave their informed consent prior to death, in compliance with local institutional and legislative requirements. All experiments were performed in accordance with relevant institutional and legislative guidelines and regulations.

**Figure 4:**
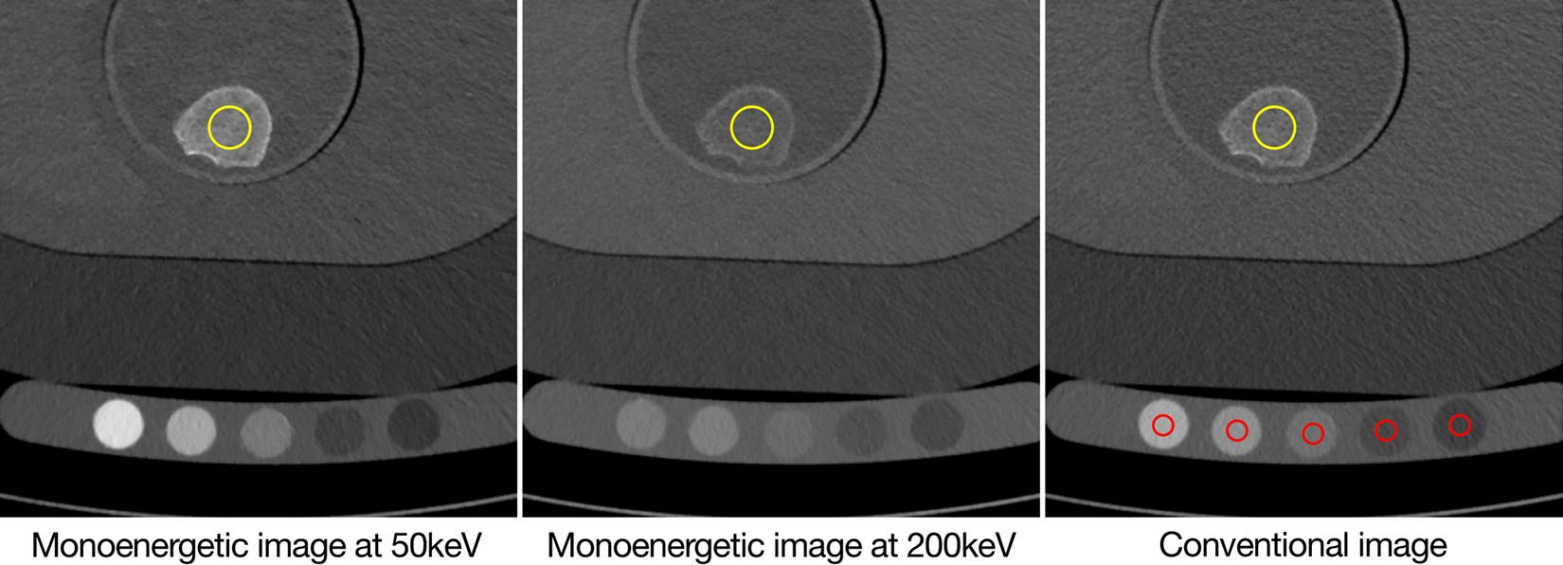
Vertebral specimens in the anthropomorphic abdomen phantom as shown in the conventional image and two different monoenergetic reconstructions. Red circles illustrate regions-of-interest (ROI) in the QCT phantom, and yellow circles illustrate ROI placement in the specimens.

Specimens consisted of mid-vertebral, 10-mm-thick axial slice of a thoracic vertebra between Th5 and Th12. Specimens were preserved in formalin, after complete removal of surrounding soft tissue. Before the scan, specimens were immersed in water bath and air inside the trabecular was eliminated using a vacuum machine. Specimens were then placed in the same anthropomorphic abdomen phantom analogous to their physiologic orientation in the human body (Fig. 4). Successively, two extension rings simulating fat were placed around the phantom, with the smaller one increasing the diameters by 350 × 250 mm, and the larger one by 400 × 300 mm, simulating patients with waist circumferences 98.5 cm and 114.3 cm, respectively.

### CT imaging protocols

Spectral CT images were acquired by using a dual-layer spectral CT scanner (IQon Spectral CT, Philips Healthcare, Best, the Netherlands).

An abdomen protocol was used for all examinations, with a fixed tube voltage of 120 kV and a pitch of 1.058. HA phantom scans were performed with different exposures of 1000, 500, 250, 125, 50 and 38 mAs; mid-vertebrae specimens were scanned with 500, 250, 125, 50 and 10 mAs. For scans with 500 mAs, tube current was 700 mA and exposure time was 709 ms. For the other exposure levels, exposure time remained the same while tube current was adjusted accordingly.

All vertebral specimens scans were performed twice, with the use of two different extension rings of the anthropomorphic simulating two degrees of obesity.

CT Dose Indices (CTDI_vol_) were recorded from the dose report generated by the scanner. Scan lengths were 50 mm per mid-vertebral specimen. Estimated effective doses (ED) were calculated directly from Dose Length Product using the k factor approach^41^. Signal- and Contrast-to-Noise-Ratio (SNR/CNR) were computed using mean and standard deviation in the homogeneous regions of the qCT calibration phantom.

We used the same reconstruction protocol for both qCT and spectral CT based BMD quantifications. Reconstruction kernel was spectral with the level set at 4, the slice thickness was 0.67 mm. The field of view was 200 mm by 200 mm with a grid size of 512 by 512 pixels. HA phantom scans at 1000 mAs were used to determine the spectral absorption behaviour of HA. The remaining scans were used to validate the BMD quantification method based on spectral information.

### BMD quantification based on spectral imaging data

For determining the spectral absorption behaviour of HA, the HA phantoms with four different known HU concentrations were scanned at 1000 mAs. Theoretically higher radiation exposure increases the number of photons, reducing image noise without changing the HA absorption nature in the spectrum. Virtual monoenergetic images at 50 (HU_*L*_) and 200 keV (HU_*H*_) were reconstructed. ROIs were manually drawn by a radiologist in the four regions in each phantom (Fig. 3). ROIs were cylinders with a diameter of 5 mm and a height of 33 mm. CT numbers (Hounsfield Units (HU)) inside the ROIs were extracted. A line function depicting the relationship with two monoenergetic HA HUs was fitted:

1 $${{\rm{HU}}}_{L}=a\cdot {{\rm{HU}}}_{H}+b$$

**Figure 5:**
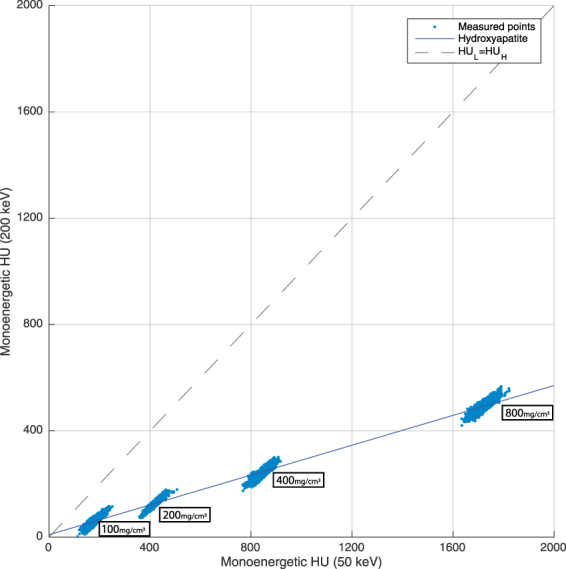
Characteristic lines of HA CT numbers of monoenergetic 50 keV and 200 keV images. Blue dots indicate pixels values within the four known concentrations of the HA phantom. The blue line is the corresponding regression line of pixels. For comparison, the dashed line illustrates the behaviour of materials with comparable CT numbers in low and high keV images, respectively (i.e., materials with low atomic numbers).

HU_*L*_ and HU_*H*_ are CT numbers for HA measured using monoenergetic 50 and 200 keV images. *a* is a fitting parameter specific for HA. *b* is a fitting parameter adjusting for water. In this set-up, pixels in the ROIs only contain HA and water, therefore pixels in the ROIs (pairs of HU_*L*_ and HU_*H*_ for HA) are always located along or close to the line described by equation (1) (blue line in Fig. 5). The higher the HU_*L*_ and HU_*H*_ are, the further of the points on the line to the origin are, the denser the HA concentration is. HU_*L*_ is proportional to the exact concentration of HA (BMD):

2 $${{\rm{HU}}}_{L}=u\cdot {\rm{BMD}}+v$$

The fitting parameters *u*, *v* were obtained from known BMD values of the four HA phantoms and the corresponding measured mean CT numbers in the monoenergetic image at 50 keV derived from the calibration scan. *u* is determined by the mAss attenuation coefficient of HA at monoenergetic 50 keV. *v* is the CT number determined by water. *a*, *b*, *u* and *v* are scanner specific variables computed once prior to specimen scans.

Next, the calibration lines from equation (1) and equation (2) were used to identify and quantitate BMD values in virtual spectral images. Scans with lower exposures (500, 250, 125, 50 and 38 mAs) were included: a measurement of two CT numbers (HU′_*H*_, HU′_*L*_) at 50 and 200 keV were extracted from the corresponding monoenergetic images. The distance d from this measurement (HU′_*H*_, HU′_*L*_) to the line as specified by equation (1) was computed:

3 $$d=\frac{|a{{\rm{HU}}}_{H}^{^{\prime} }-{{\rm{HU}}}_{L}^{^{\prime} }+b|}{\sqrt{{a}^{2}+1}}$$

We applied plain thresholding (*d*) to identify HA. A measurement was considered bone if it is closer to the regression line as defined in equation (1), i.e. *d* was less than 20 HU. If *d* was greater than 20 HU, the measurement was likely to represent non-HA material, such as soft tissue. For each measurement of (HU′_L_, HU′_H_) that was considered to represent bone, its BMD value was estimated. In order to minimize possible measurement errors, each measurement was projected to the HU_L_/HU_H_ regression line as specified in equation (1). For this, the closest point on the line described with equation (1) (HU_L_, HU_H_) to the actual measurement (HU′_L_, HU′_H_) was determined with the following equation:

4 $${{\rm{HU}}}_{L}=\frac{{{\rm{HU}}}_{L}^{^{\prime} }+a{{\rm{HU}}}_{H}^{^{\prime} }-ab}{{a}^{2}+1}$$

The resulting HU_L_ was finally converted to a BMD value with calibrated equation (2). This procedure was performed pixel-by-pixel in a given ROI, and resulting BMDs were averaged.

Analogously, equations (3) and (4) were used to determine HA content and equation (4) to quantitate BMD values for vertebral specimens.

### BMD quantification in vertebral specimens based on qCT

ROIs were placed manually by a radiologist in the centre of vertebral specimens. These ROI were 2D circles with diameter 5.9 mm or 7.8 mm, depending on the vertebra size, and were placed on the 3 slices in the middle of the vertebrae (Fig. 4).

For comparison purpose, BMD was also calculated with the conventional qCT method (QCT Pro Bone Mineral Densitometry Software, Phantom Module. Version 4.0, Mindways, TX, USA). For these scans, a calibration phantom (Mindways) was placed beneath the anthropomorphic phantom containing 5 rods with known density equivalents for phosphate and water. For each scan, mean values of the five rods in the QCT phantom were measured in the conventional images generated by the dual-layer spectral scanner. The ROI selection was similar to the one described in the previous section (Fig. 4). Conversion functions for the scanner were computed in a least square manner with MATLAB.

### Statistical analysis

To describe measurement errors for the quantification of known HA concentrations based on spectral information, descriptive statistics were used containing absolute values and relative values compared to the known concentration as given by the manufacturer of the phantom. Descriptive statistics were also used for describing BMD measurements in vertebral specimens both spectral-based and using qCT with different exposures and different setups simulating different degrees of obesity. Normality of BMD measurement values was confirmed by Shapiro-Wilk tests.

To assess correlations between qCT and spectral CT measurements, both Pearson and Spearman correlations were used. Since Pearson’s r was consistently higher than Spearman’s ρ, linear correlations were assumed. Agreement between BMD measurements based on spectral CT and qCT was assessed with Bland-Altman plots^42^, and paired-samples t-tests were used to assess values for statistically significant differences.

In order to assess the influence of different degrees of simulated obesity by using extension rings around the abdomen phantom, multivariable linear regression models were performed with BMD based on spectral data as dependent and BMD based on qCT and simulated degrees of obesity as independent variables, separately for different mAs. Differences between values from two setups simulating two different degrees of obesity were compared by using paired-samples t-tests.

Statistical analyses were performed with SPSS 24 (IBM; Armonk, NY, USA), with two-sided P < 0.05 indicating a significant difference.
